# Supplementary material for: Characteristics of ocular-related emergency department visits: Five-years data from a tertiary care center in Riyadh, Saudi Arabia
Source: PLoS One. 2024 Nov 11;19(11):e0310179. doi: 10.1371/journal.pone.0310179 (PMC11554231; doi:10.1371/journal.pone.0310179)
Supplement: Attachment — Submitted filename: Response to Reviewers.docx [file pone.0310179.s001.docx]

Dear Editor,

On behalf of the authors, I would like to thank you for considering our manuscript for publication in the PLOS ONE journal. We would also like to thank all reviewers for offering their valuable time and effort to review our paper. The reviewers’ comments and feedback are very helpful, and we hope our responses will have improved the paper.

**Reviewers’ Comments:**

**Reviewer 1 comments: Well written retrospective epidemiological study relevant to the local region of Saudi Arabia.**

**Our response:** Thank you for your insightful feedback and positive comments.

**Reviewer 2 comments: The study is comprehensive and well done**

**Entry and exit criteria and how to collect samples are well explained.**

**The text tables well represent the statistics obtained from the analyses.**

**In the discussion section, the cases have been well compared and analyzed with other studies.**

**As a suggestion, it is better to give more details in cases with significant statistics that are the same or different from the rest.**

**Our response:** We appreciate your positive comments and feedback about our study. We agree with your suggestion, and we added the statistics of the studies in the discussion section that either the same or different from the rest. Please revise the manuscript file.

Thank you all again for your comments and feedback. We hope that we have addressed all comments to improve our paper.

If you have any other questions, please do not hesitate to ask.

Yours sincerely,

Dr. Abdullah Alshibani

Assistant professor in Emergency Medical Services
